# Rare Fungal Infection Linked to a Case of Juvenile Arthritis

**DOI:** 10.7759/cureus.3229

**Published:** 2018-08-29

**Authors:** Karin Ried, Peter Fakler

**Affiliations:** 1 NIIM Research, National Institute of Integrative Medicine, Melbourne, AUS

**Keywords:** juvenile arthritis, systemic fungal infection, aspergillus, sagenomella

## Abstract

Juvenile arthritis with unknown disease etiology is also known as juvenile idiopathic arthritis. Symptoms include joint pain, swelling, and stiffness, and standard treatment involves immunosuppressant medication.

Here we present a case of juvenile idiopathic arthritis with severe malnutrition and worsening of symptoms, which restrained a nine-year-old girl to a wheelchair with minimal movement capacity and low energy during standard immunosuppressant therapies over the course of three years.

Our innovative Pathogen Blood Test combining cytology-based microscopy and genetic analysis using a pan-fungal primer assay and sequencing identified a systemic fungal infection with *Sagenomella* species, closely related to *Aspergillus*, and a soil-dwelling highly pathogenic fungus, which had previously been linked to a fatal veterinary case of arthritis and malnutrition.

Our test results encouraged a radical change of the patient’s treatment plan, including cessation of the regular immunosuppressants, including steroids, over six months. The patient made a progressive recovery, including complete reversion of the previously swollen and painful joints, development of a good appetite, and return to liveliness. Within the year of change from immunosuppressants to immune-supportive integrative nutritional therapies, including regular intravenous vitamin C, and oral vitamin D, as well as gentle aqua- and physiotherapy, the patient started to gain weight including muscle mass and regained strength and movement in the hands, arms, and legs. She was able to walk again within 18 months. Her mood and energy levels continued to improve and she was able to return to school full-time.

## Introduction

Juvenile idiopathic arthritis (JIA), formerly known as juvenile rheumatic arthritis, juvenile chronic arthritis, or pediatric rheumatic disease, is the most common type of arthritis in children under the age of sixteen. JIA is an inflammatory condition, causing persistent joint pain, swelling, and stiffness, affecting 1 in 1000 children in Australia and other Western countries. It has a global incidence rate of 7-23 cases per 1000 patient-years [1-3].

While severe physical disability experienced by 80% of JIA cases may resolve over time in half of the cases, long-term active arthritis resulting in permanent disability is experienced by 43% of JIA cases [4]. Long-term unresolved JIA often results in joint deformity and destruction—with 50% requiring orthopedic surgery for joint replacement—postural abnormalities, muscle atrophy, and weakness as well as psychological issues [4].

A higher mortality rate in children with systemic juvenile idiopathic arthritis (5/1000 patient-years) compared to their age-matched peers has been observed (1.6/1000 patient-years) (Poster abstract: Davies R, Southwood T, Kearsley-Fleet L, Lunt M, Hyrich K. Standardised Mortality Rates are Increased in Patients with Severe Juvenile Idiopathic Arthritis. EULAR Annual European Congress of Rheumatology; 2014, 73:588.1; Abstract no. FRI0557. https://ard.bmj.com/content/73/Suppl_2/588.1. DOI 10.1136/annrheumdis-2014-eular.3241).

The goal of therapy for systemic JIA focuses on the prompt control of active inflammation and symptoms and the prevention of a number of disease- and/or treatment-related morbidities such as growth disturbances, joint damage, and functional limitations [1]. Recommended medications include non-steroidal anti-inflammatory drugs (NSAIDs), glucocorticoids, and immunosuppressants, such as anakinra (interleukin-1 receptor blocker), methotrexate (dehydrofolate reductase inhibitor), adalimumab (TNF-alpha blocker), leflunomide (pyrimidine synthesis inhibitor), and rituximab (monoclonal antibody) [1].

An improvement of symptoms is to be expected in about 36% of cases without medication, while treatment with methotrexate over six months can increase the improvement rate by about 30% to 63% [5]. Treatment with corticosteroids may help in a third (32%) of cases within 12 months, while a combination of steroids and methotrexate only slightly increased response rate to 37% [6].

Newer therapies with TNF-alpha blockers and interleukin blockers may achieve a 25-50% effectiveness when used early in the disease for a defined time period [2]. However, follow-up studies reporting on effects of any long-term drug therapies indicated for JIA are sparse.

The case of juvenile idiopathic arthritis described in this report was treated in accordance to JIA guidelines over three years, however, with symptoms worsening over time. The causality of the condition had not been established, and the diagnosis was hampered by the patient’s family history of autoimmune conditions, including lupus and Crohn's disease, pointing towards a potential genetic component.

## Case presentation

A nine-year-old girl presented to the National Institute of Integrative Medicine (NIIM) Clinic in Melbourne, Australia, in June 2016 with chronic pain, extreme muscle wasting requiring a wheelchair, growth retardation, severe underweight (20 kg), swollen and painful joints, heart palpitations, loose stools, and headaches. Her condition and extreme weakness didn’t allow her to move her limbs without assistance; she was not able to feed herself, or move her legs without assistance or stand up.

The nine-year-old had not been able to attend school for several months due to the severity of her illness, and she had been in and out of hospital on a regular basis. The girl had been diagnosed with juvenile idiopathic arthritis (JIA) three years earlier and had been treated with standard medications for the potential autoimmune condition, including regular corticosteroid infusions with methylprednisolone, treatment with methotrexate, and anakinra, a recombinant and modified interleukin-one-receptor-antagonist.

Despite these conventional treatments, her condition had progressively worsened over the course of three years, and by the time she presented to the NIIM clinic her prognosis was extremely critical. The sudden onset of illness three years earlier with extremely high fever and rashes, coincided with the girl’s pet dog’s illness, sudden death and exposure to the dog’s blood into the girl’s eyes. Her pet dog had been ill with a wobbly walk, weight loss, and listlessness prior to its accidental death with open wound blood loss, suggesting a plausible path of infection.

Materials and methods

Our research lab at the National Institute of Integrative Medicine (NIIM) in Melbourne, Australia has developed a two-part Pathogen Blood Test assay combining cytological microscopy and genetic analysis of the pathogen by polymerase-chain-reaction (PCR) DNA analysis [7]. Fresh and processed blood is handled in air-filtered laboratory cabinets with sterilised equipment.

In the first part of this Pathogen Blood Test, the microscopic analysis, 10 ml of the patient’s blood is treated with a saponin-enriched buffer to lyse the red blood cells [8]. The treated blood is vacuum-filtered through a special polycarbonate filter with eight micron holes and stained with standard May-Gruenwald-Giemsa for cytological analysis using a Leica light microscope (Leica Microsystems Inc., IL. USA) with 63x10 ocular magnification. The image is captured with the Leica EC3 digital camera (Leica Microsystems Inc., IL. USA). This first part of the Pathogen Blood Test of isolating rare cells in blood by filtration has been adapted from the cytology-based Circulating-Tumour-Cells (CTC) Isolation-by-Size-of-Epithelial-Tumour-cell (ISET) technology developed by Rarecells, France, which focuses on the isolation and identification of human cancer cells from blood [9].

Using light microscopy we screen the erythrocyte-free enriched blood for human and non-human cells, including potentially pathogenic single or multicellular organisms, such as bacteria, protozoa, parasites, or fungal elements.

**Figure 1:**
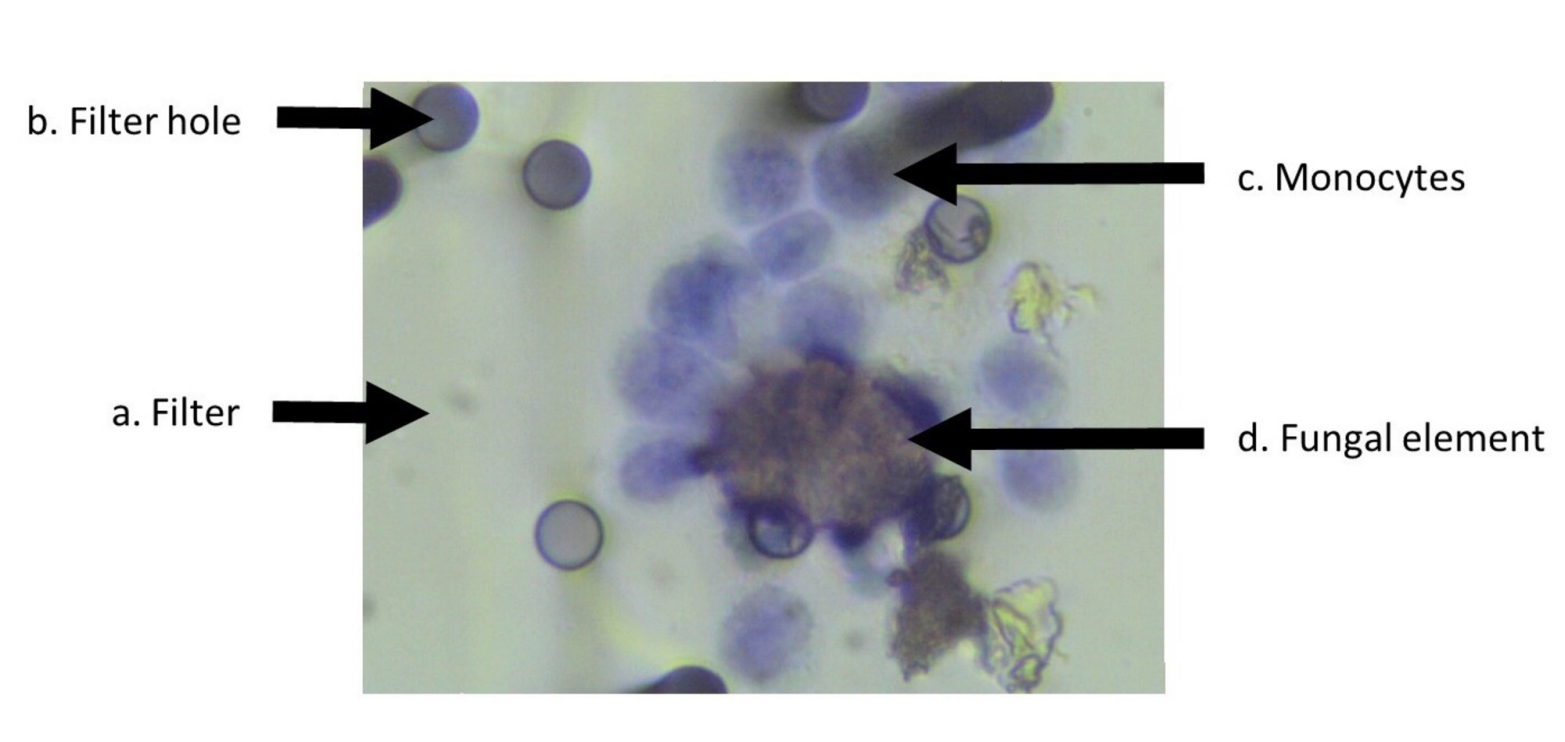
Microscopy photograph of stained filter with erythrocyte-free blood from patient with juvenile arthritis The filter (a) has 8 micron (micrometre) pores (b) (black circles) and is shown to have retained human immune cells in the form of monocytes (c) of 8-12 microns (blue stained) and fungal elements (d) (brown stained), the main central fungal element (d) shown being around 30 microns.

In this case study, the microscopy revealed the presence of a large number of fungal elements amongst inflammatory cells (Figure 1).

In the second part of the Pathogen Blood Test, any non-human organisms detected in the processed blood can be further identified by genetic analysis [7].

As we found an unusually large number of fungal elements in the microscopic analysis in this case, we proceeded with fungal DNA analysis as follows:

Genetic identification of fungal elements was adapted from references [10] and [11]. DNA was extracted from 3 ml whole blood using a bead beating step to lyse tough-walled cells including fungal spores and hyphens with MP Biomedical FastPrep®-24 Instrument and Lysing Matrix B (www.mpbio.com) (MP Biomedicals, LLC., CA, USA), followed by Qiagen DNeasy tissue protocol using the Qiagen blood and tissue DNA extraction kit (www.qiagen.com) (Qiagen, Hilden, Germany). Subsequently, a pan-fungal PCR-assay with primers and PCR conditions as described in [10] was performed, using a real-time qPCR Roche Light-cycler-1.5, (Basel, Switzerland). The PCR products were purified with the Qiagen PCR cleaning kit and Sanger-sequenced with the same primers by the Micromon DNA Sequencing Facility at Monash University, Melbourne, Australia. The acquired sequence was then analyzed by comparative genetic analysis using the Basic-Local-Alignment-Search-Tool (BLAST) search tool (https://blast.ncbi.nlm.nih.gov/Blast.cgi) [12].

The amplified fungal DNA best matched *Sagenomella *species of the ascomycete family Trichocomaceae, which also includes *Aspergillus *and *Penicillium*.

**Figure 2:**
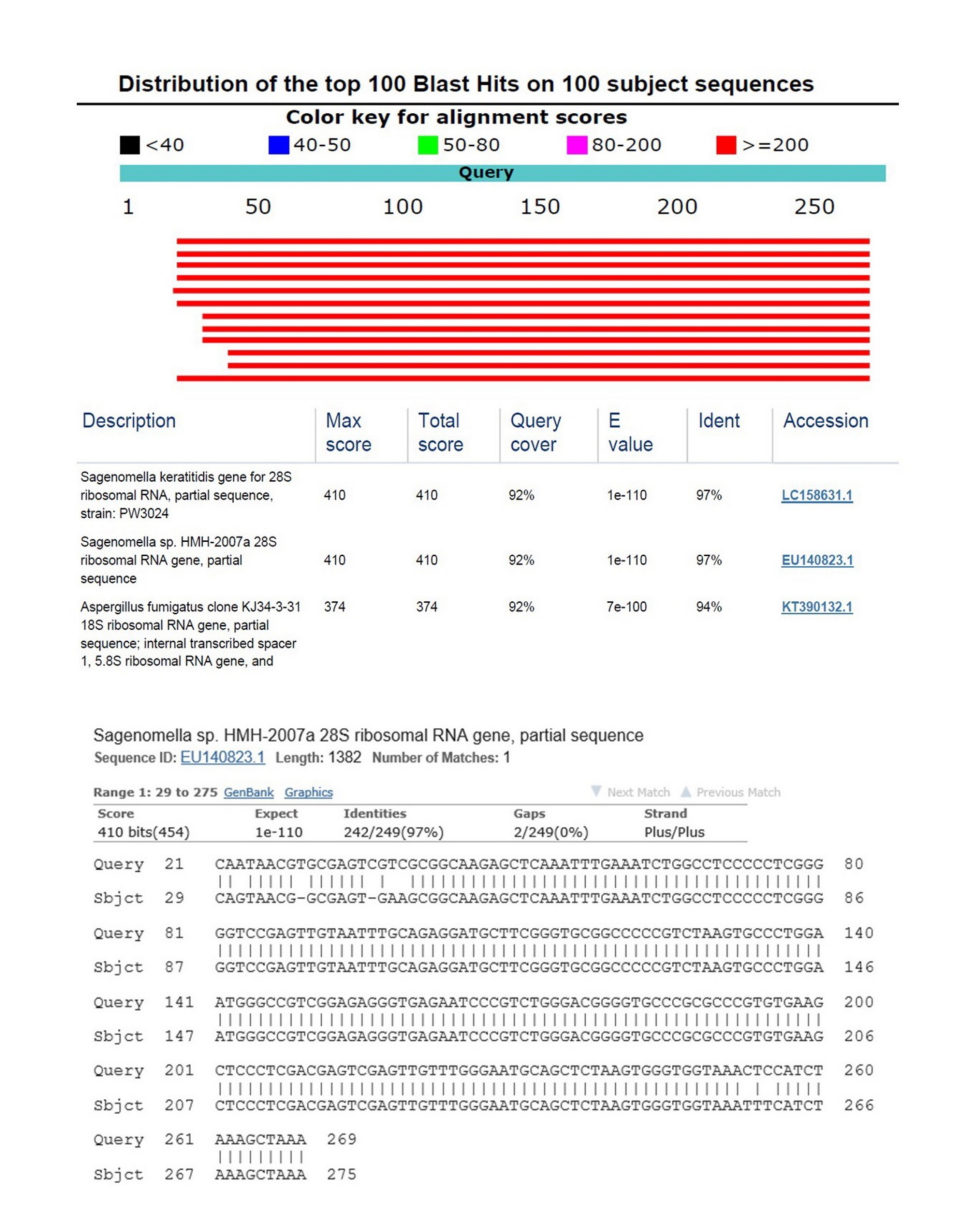
Fungal DNA sequence extracted from the patient's blood and BLAST comparison with 97% identity to Sagenomella species DNA - deoxyribonucleic acid, BLAST - basic local alignment search tool, Query - sequence derived from case, Sbjct - nucleotide sequence in BLAST database. First three BLAST hits are listed and comparison of sequence between query to Sagenomella species with query cover of 92% and sequence identity of 97%.

Sequence identity of the pan-fungal PCR-product to *Sagenomella *and *Aspergillus *species was high (97% and 94%, Figure 2). 

Pathogenic infections with *Sagenomella *have been described in the literature, including a fatal systemic fungal infection in a dog with a history of listlessness, extreme weight loss, joint issues, and multiorgan involvement, as identified in the autopsy report [13]. The girl’s dog in this case report had also displayed similar symptoms before sudden death, and transmission of an infection appears plausible as the sick dog’s blood came in contact with the girl’s eyes according to her mother.

Prompted by these findings, we tested our patient for Aspergillus antibodies and found highly elevated serum levels of immunoglobulin E (IgE) = 8.7 kU/L (cat III; high), while the absence of elevated IgM serum levels suggested a chronic rather than an acute infection.

Our test results encouraged a radical change of the patient’s treatment plan, by which immunosuppressive therapy, which at that time consisted of prednisolone injections, was withdrawn gradually over six months. At this time the patient was too weak for any anti-fungal medications. Instead, immune system supportive treatments were provided, including immune-stimulating herbs and nutrients, such as high dose vitamin C, vitamin D, and ozone therapy [14, 15]. In addition, the patient was able to participate in gentle water-based physiotherapy sessions two-three times a week.

Over the next six months, the patient made a progressive recovery, including remarkable reversion of the previously swollen and painful joints, with only occasional pain in the wrist. Importantly, the patient’s appetite, energy levels, and mood improved. She returned to being able to use her hands again to feed herself and participated in regular walking exercises in water. After about six months since cessation of the treatment with regular immunosuppressant medications, the patient started to gain weight (>20kg) including muscle mass and had solid bowel movements. Her mood and energy levels continued to improve and about ten months after detection of the systemic fungal infection and the beginning of the new treatment program, she was able to return to school again firstly part-time then full-time.

A repeat Pathogen Blood Test one year after the initial test identified remaining fungal presence with *Sagenomella*, albeit to a lesser extent than initially. The ratio of fungal elements to immune cells determined by microscopy in June 2016 was 1/7, and at the time of the repeat testing in May 2017 the ratio was 1/20.

The repeat blood test also identified an increased number of Circulating Tumour Cells (CTC) from 0.1 CTC/ml in June 2016 to 12.6 CTC/ml in May 2017 associated with an increased risk of malignancy [15], in line with findings of higher malignant potential in JIA in the literature [16].

Additionally, the patient had severe anemia with thrombocytosis, high copper, low urea, and high creatinine, prompting an intravenous iron infusion as per protocol for children, which the patient tolerated well.

As a result of the remnant findings of fungal elements in her repeat blood test in May 2017 and the patient being stronger than one year prior, the patient was prescribed a 60-day course of anti-fungal medication of itraconazole.

Monthly intravenous vitamin C infusions of 15-30 grams per dose continued over the next year, as well as regular immune-stimulating nutrients, including vitamin D, B-vitamins, zinc, magnesium, and glutathione. To date, about two years after undertaking the first Pathogen Blood Test, the patient has continued to improve, has been attending school full-time, and has been able to walk unassisted for short distances.

## Discussion

The case of juvenile idiopathic arthritis described in this report was treated in accordance to JIA guidelines over three years, however, with worsening symptoms. The causality of the autoimmune condition had not been established, and the diagnosis was hampered by the patient’s family history of autoimmune diseases.

The innovative Pathogen Blood Test described in this report established a possible link between the patient’s sudden onset and extreme arthritic condition and the rare soil-dwelling, opportunistic, and potentially pathogenic fungal infection associated with systemic organ distribution including joint involvement, as well as extreme disturbances in nutrition absorption, weight loss, and anorexia.

Previously, the systemic fungal infection *Sagenomella chlamydospora* had been responsible for a fatal infection in a dog, which had a history of depression, weight loss, anorexia, listlessness, and slight spasticity in the walk [13]. Postmortem examination from lesions in numerous organs, including kidneys, mitral heart valve, abdominal aorta, and vertebral discs, revealed systemic fungal infection with *Sagenomella *species, closely related to *Aspergillus *species [13]. The genus  *Sagenomella* has been described pathogenic in animals and also in humans, albeit in very rare cases [17].

The exposure to the patient’s ill dog’s blood at the time of the sudden onset of the disease supports the infection and disease etiology, however, they cannot be proven retrospectively.

The association between systemic fungal infections and inflammatory conditions such as arthritis has been described previously and can include common moulds such as *Aspergillus*, *Coccidioides*, and *Cryptococcus *[18].

Identification of systemic *Aspergillus *infections, however, is difficult to establish through culturing. *Aspergillus *does not grow or replicate in whole blood cultures; however, *Aspergillus *DNA can be detected in leukocyte pellets from the blood [11, 19].

The fact that cessation of standard immunosuppressive therapy in this case provided a quick turn-around of symptoms, supports a causal relationship between infection rather than auto-immune condition and the patient’s JIA. How much of the immuno-supportive therapies contributed to the patient’s recovery cannot be established. However, it is clear that the non-standard treatment approach was beneficial in this case.

## Conclusions

Here, we report a case of sudden onset arthritic symptoms including severe joint swelling and pain diagnosed as juvenile idiopathic arthritis, which failed to respond to standard therapy with immunosuppressant medication over three years, leading to the patient’s deterioration including extreme muscle wasting requiring a wheelchair, anorexia, low mood, and extreme fatigue.

Our innovative advanced Pathogen Blood Testing combining microscopy and genetic analysis demonstrated the presence of fungal elements in the blood identical to those previously reported to be pathogenic in a dog. These facts, along with the history of exposure to the blood of the case’s ill dog, support an infectious etiology.

Prompted by our findings, withdrawal from standard immunosuppressive therapy and institution of immuno-supportive measures, including intravenous vitamin C, vitamin D supplementation, ozone therapy, followed by short-term anti-fungal therapy led to the patient’s remarkable recovery and continuous improvement. The regaining of strength, energy and functionality, restored nutritional absorption, and cessation of inflammatory processes and arthritic symptoms, including the absence of pain and swelling of joints, demonstrated recovery.

In summary, this case demonstrates that juvenile idiopathic arthritis (JIA) may have an infectious etiology in some instances, evident by the patient’s worsening symptoms on standard immunosuppressive therapies, but response to immuno-supportive therapies. All cases of JIA may benefit from investigations into a possible infectious etiology. In those cases of JIA with systemic infectious etiology, standard immunosuppressive therapies may be ineffective.
